# Supplementary material for: Combustible ice mimicking behavior of hydrogen-bonded organic framework at ambient condition
Source: Nat Commun. 2020 Jun 19;11:3124. doi: 10.1038/s41467-020-16976-1 (PMC7305155; doi:10.1038/s41467-020-16976-1)
Supplement: Supplementary file 1 — Supplementary Information [file 41467_2020_16976_MOESM1_ESM.pdf]

Combustible ice mimicking behavior of hydrogen-bonded organic framework at ambient condition

Wang, et al.

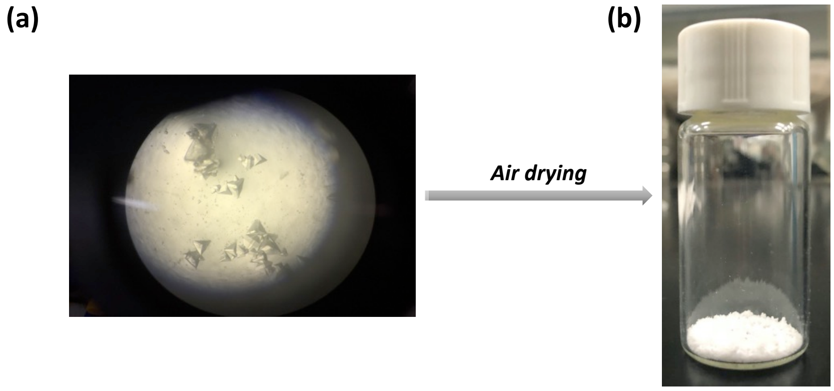

Supplementary Figure 1│Digital images of as-synthesized samples. a, Freshly prepared Gd-B single crystal. **b,** Air-dried Gd-B white powder.

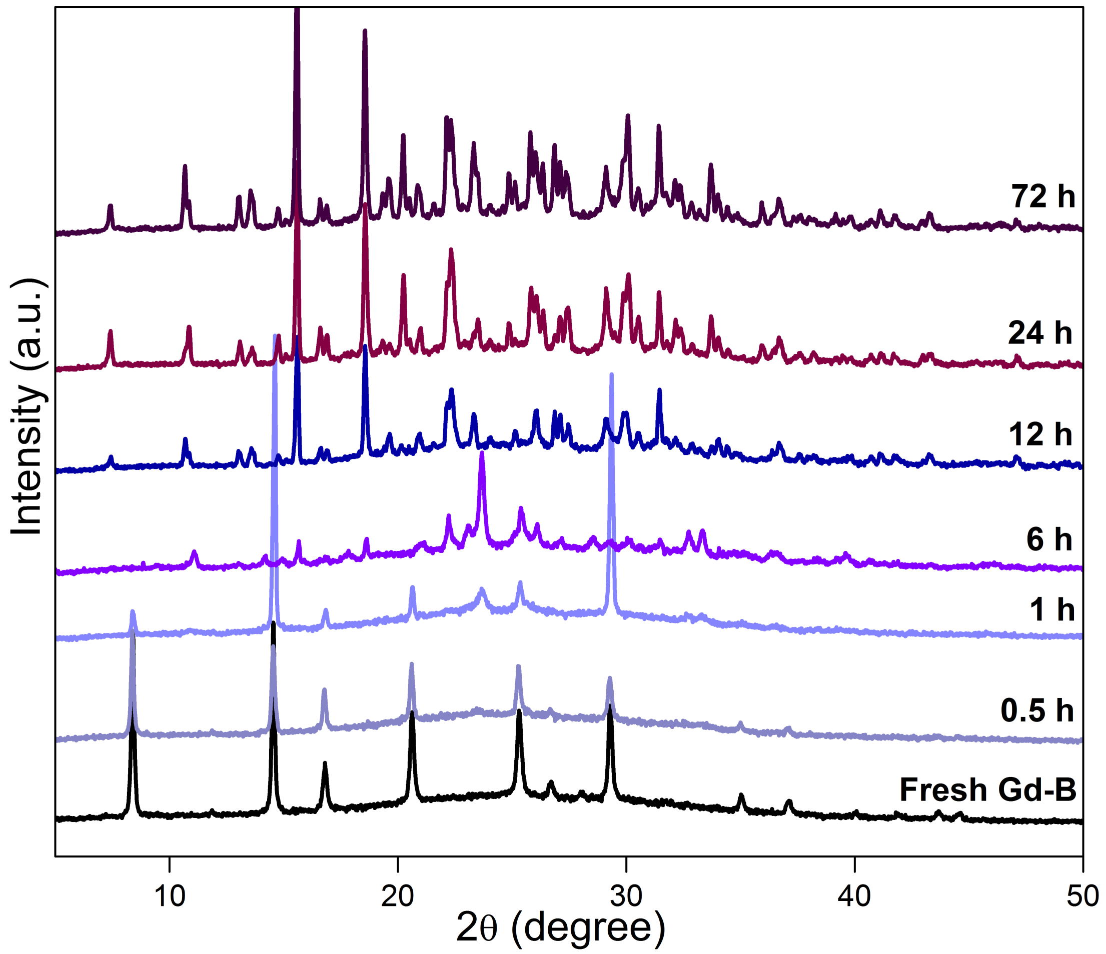

Supplementary Figure 2 | PXRD patterns of fresh Gd-B dried in air for different time.

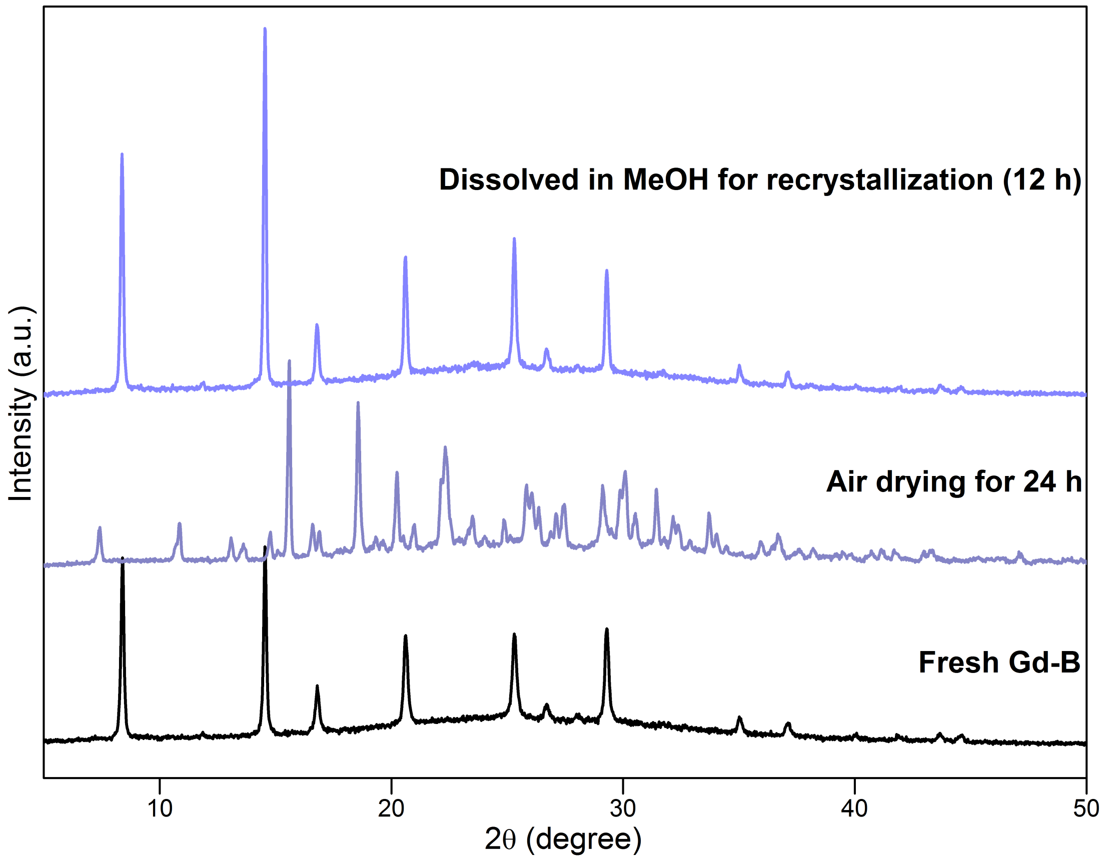

Supplementary Figure 3 | PXRD patterns of fresh Gd-B that underwent air drying for 24 h, followed by dissolving the air-dried Gd-B and recrystallizing in MeOH for 12 h.

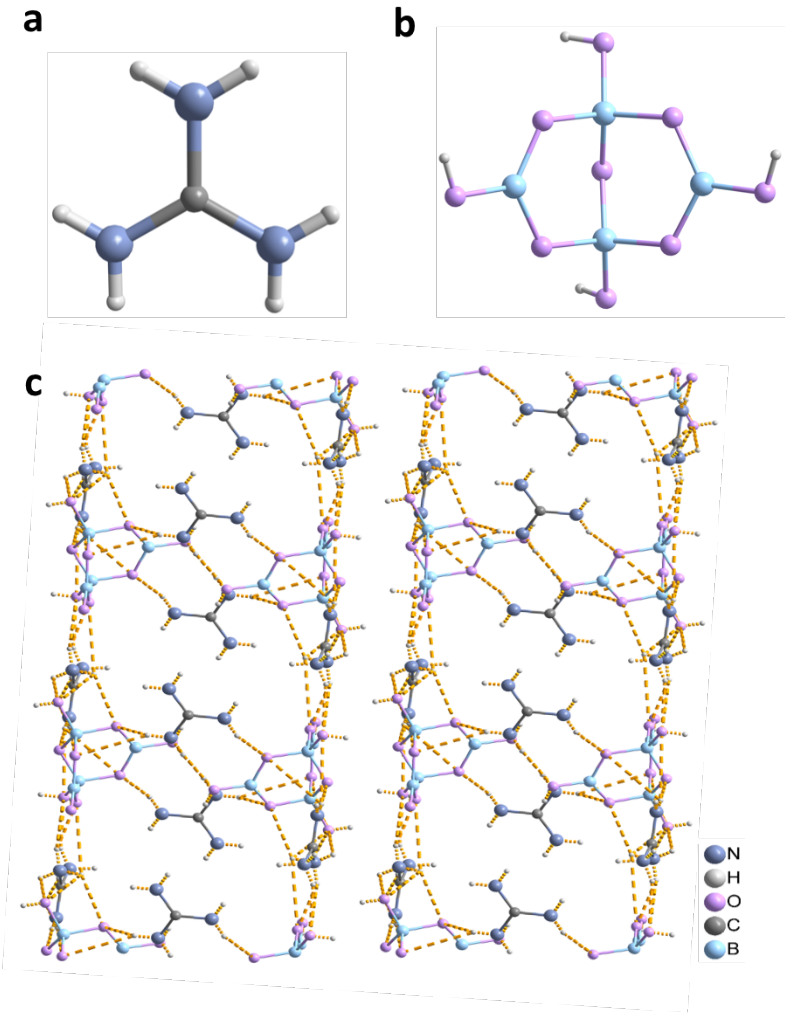

Supplementary Figure 4 | Crystal structure of guanidinium tetraborate dihydrate. **a,** guanidinium cation; **b,** $[B_4O_5(OH)_4]$ anion; **c,** $[C(NH_2)_3]_2[B_4O_5(OH)_4]•2H_2O$.

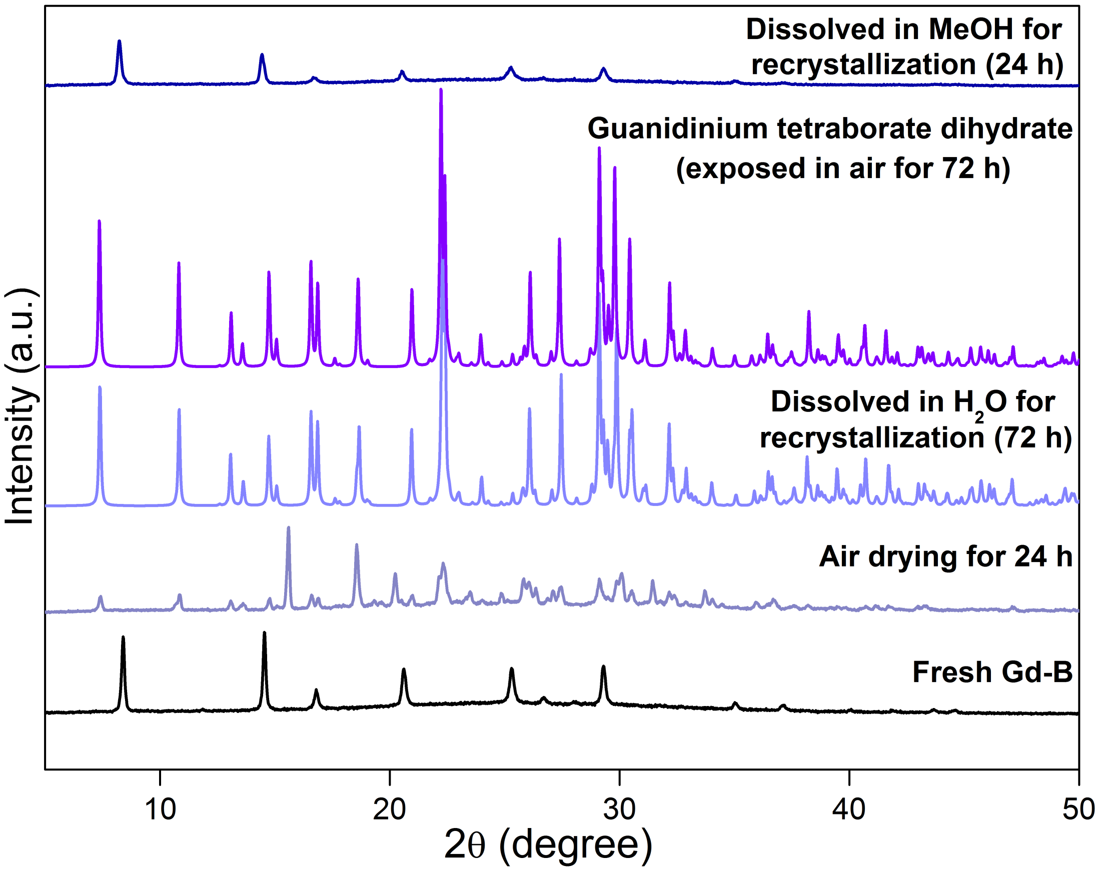

Supplementary Figure 5 | PXRD patterns of fresh Gd-B underwent air drying for 24 h, followed by dissolving the dried powder and recrystallizing in H_2O for 72 h, the obtained guanidinium tetraborate dihydrate was then dissolved in MeOH for recrystallization (24 h).

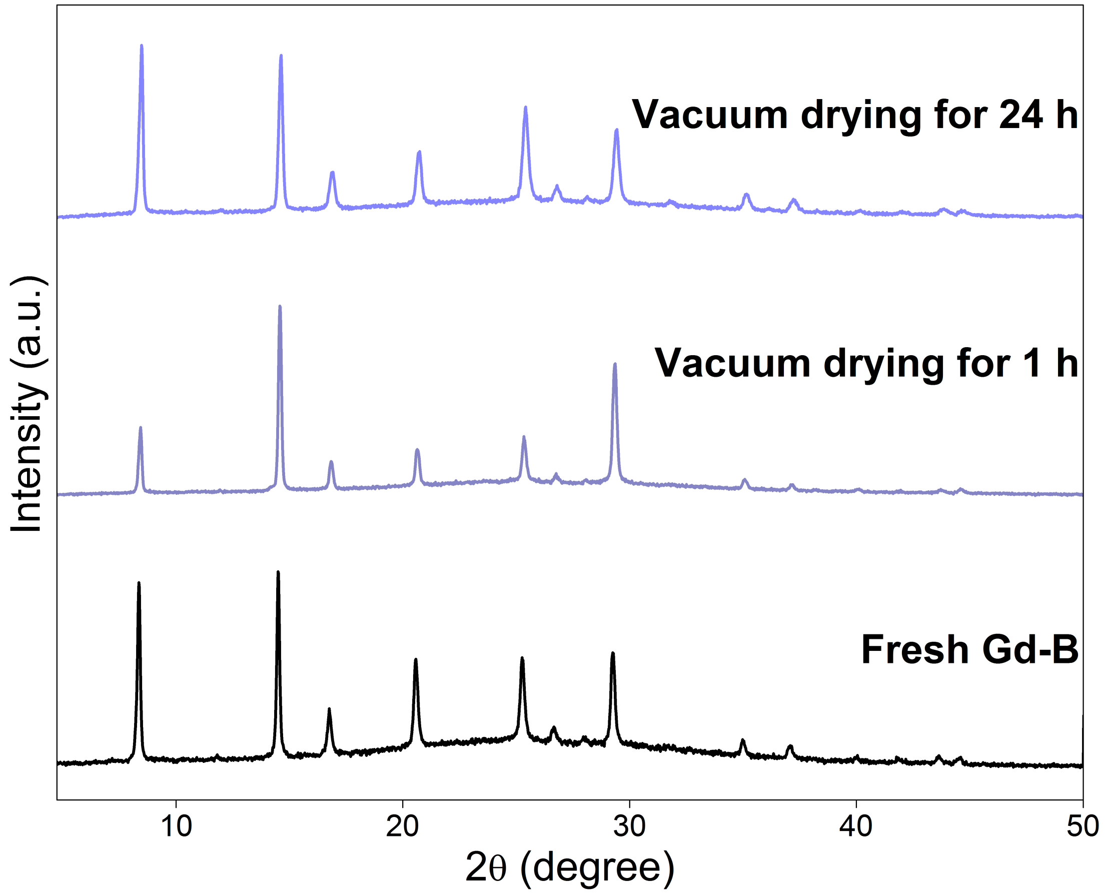

Supplementary Figure 6 | PXRD patterns of fresh Gd-B under vacuum drying for different hours.

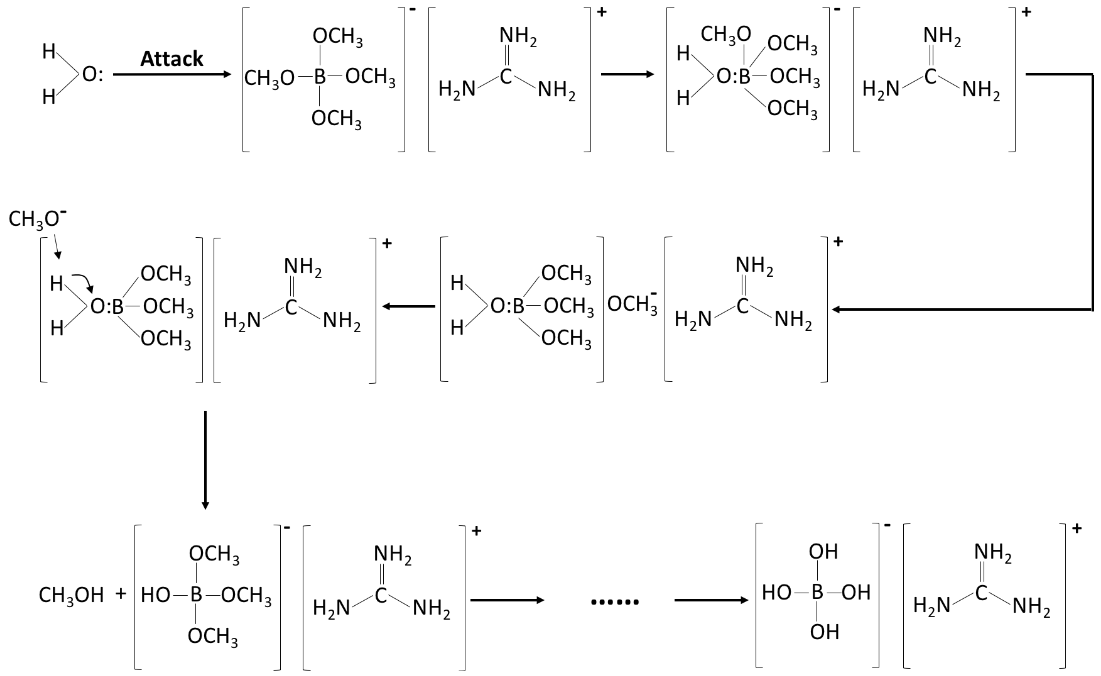

Supplementary Figure 7│ Hydrolysis mechanism of Gd-B exposing in air atmosphere. Fresh Gd-B hydrolyzes gradually upon water attack in air, accompanied with the formation of methanol that further volatilizes in air. Prolonged exposure in air lead to the complete hydrolysis of Gd-B, in which $[B(OCH_3)_4]$ totally transforms into $[B(OH)_4]$.[1]

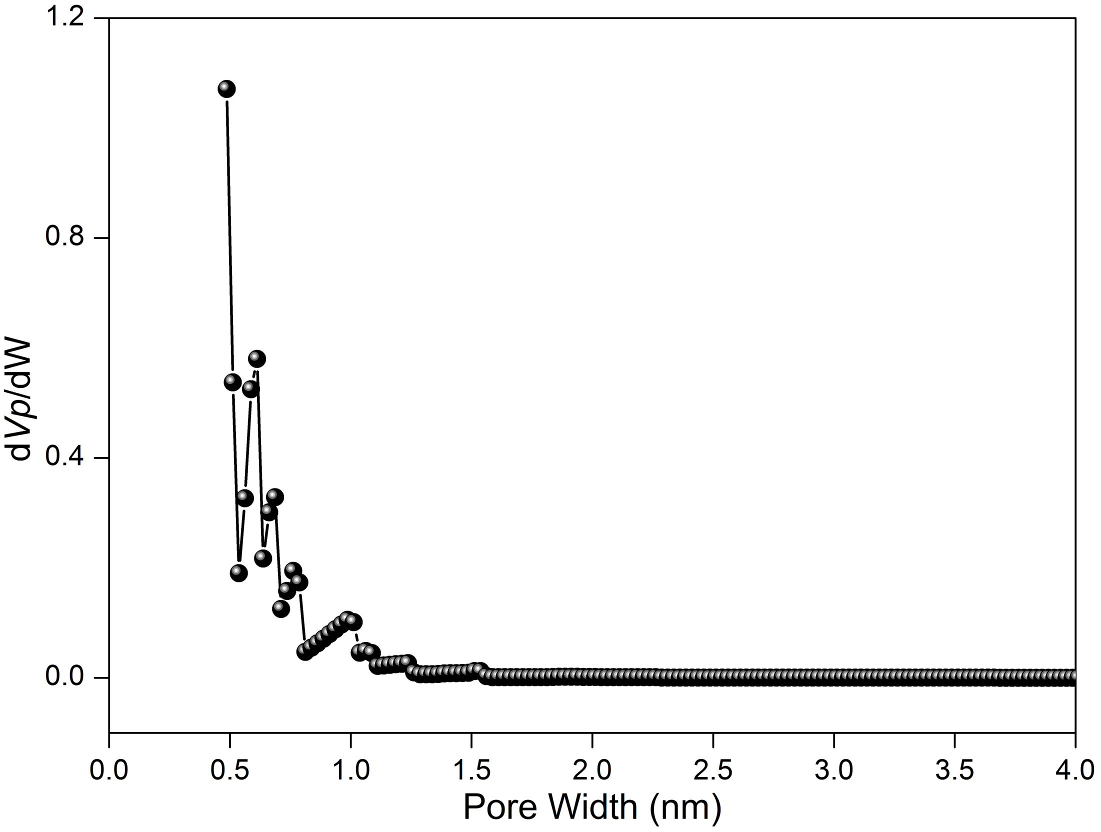

Supplementary Figure 8 | Pore size distribution of fresh Gd-B.

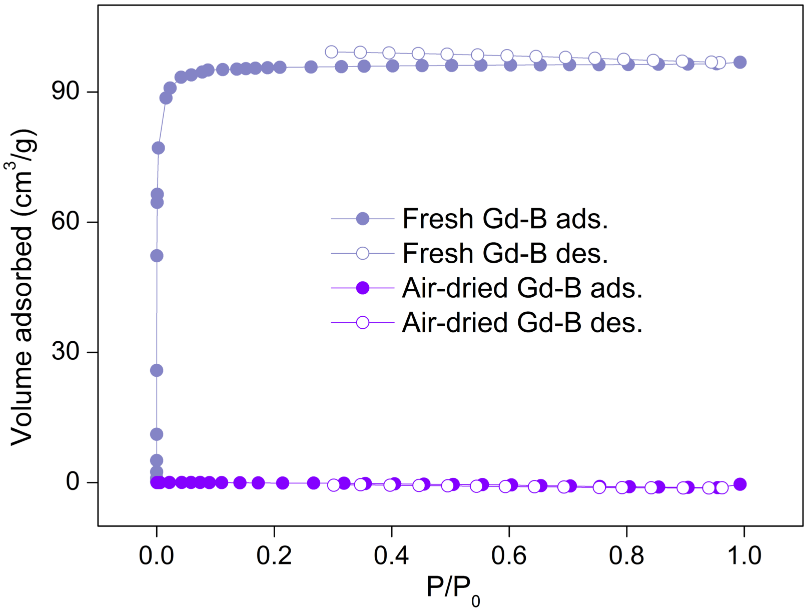

Supplementary Figure 9 | N_2 sorption isotherms of fresh Gd-B and air-dried Gd-B recorded at 77 K.

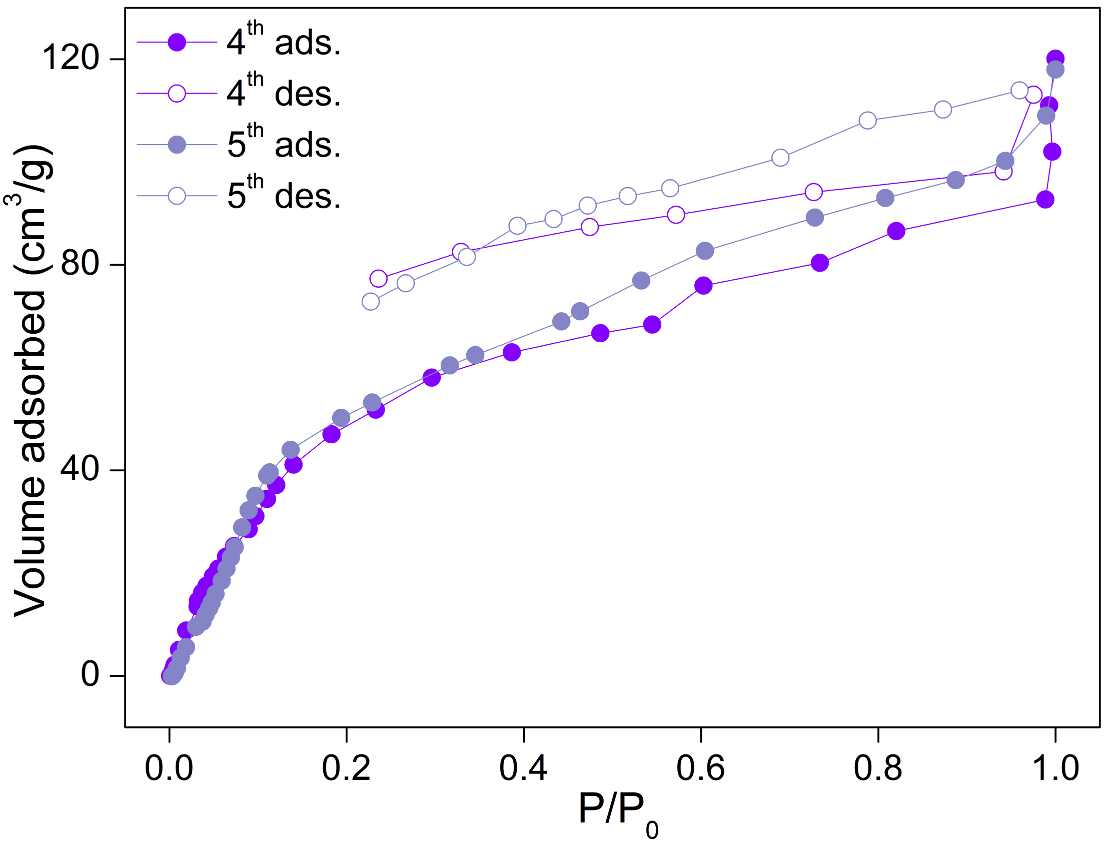

Supplementary Figure 10 | The 4th and 5th runs of MeOH sorption test over air-dried Gd-B sample at 298 K.

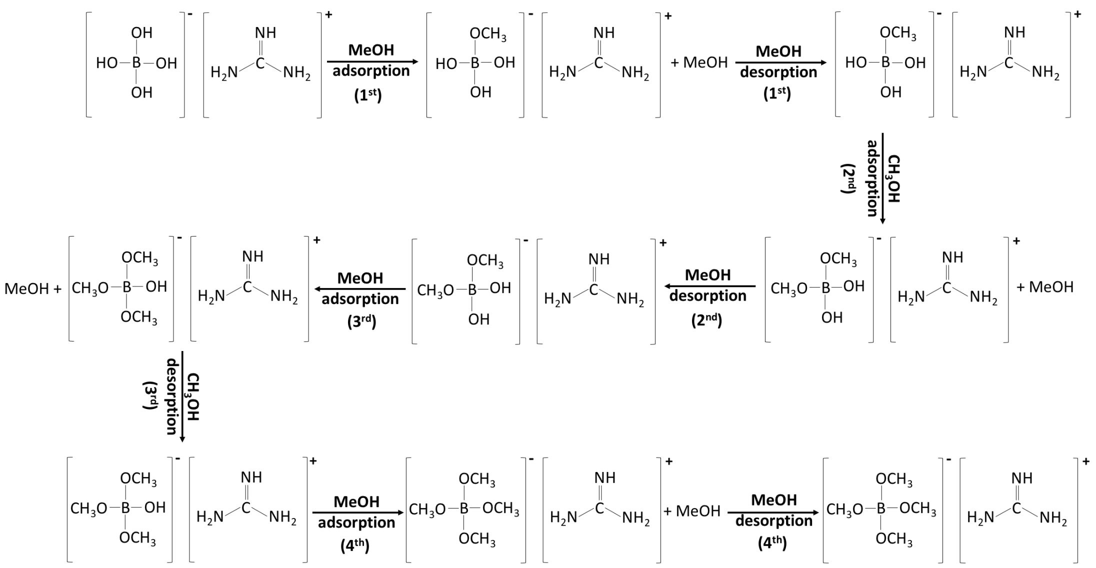

Supplementary Figure 11 | The proposed restoration mechanism of Gd-B. Note that although Gd-B framework can only be maintained after four runs of sorption tests. Specifically, air-dried Gd-B would undergo four runs of MeOH sorption tests to realize the complete restoration of Gd-B framework.

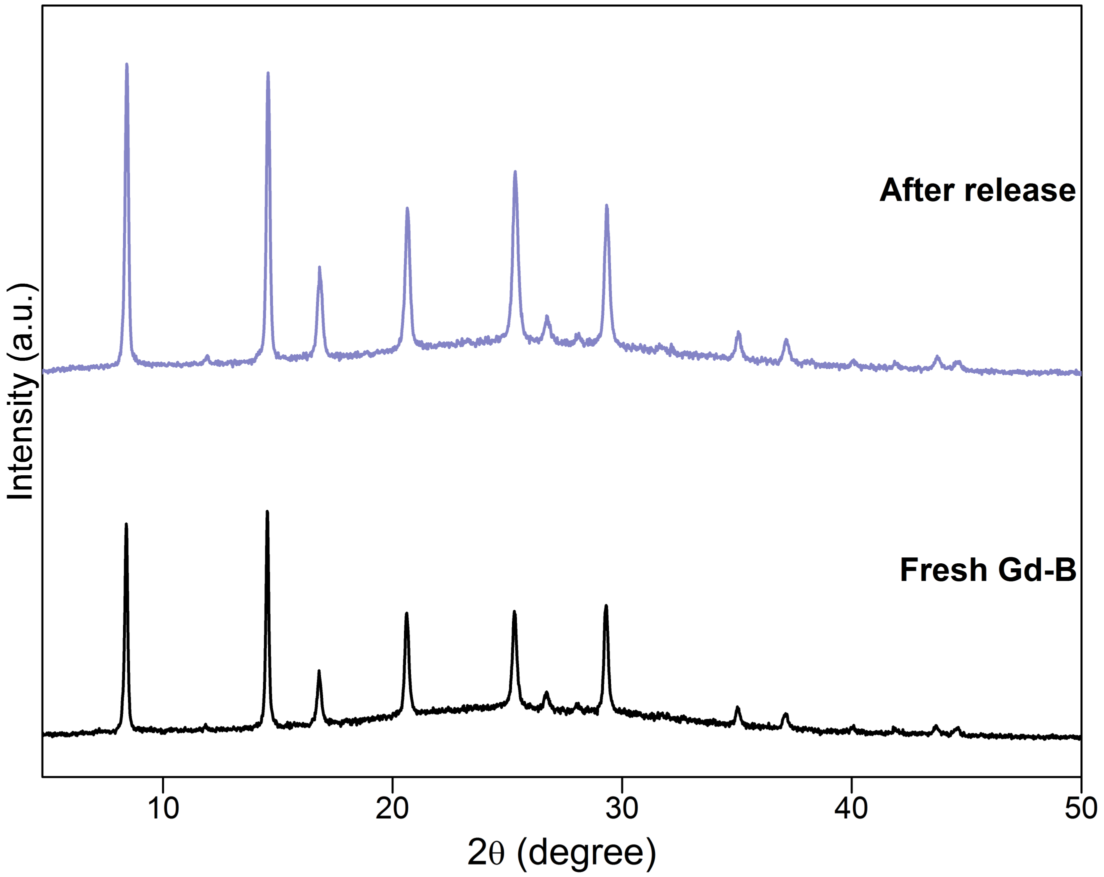

Supplementary Figure 12 | PXRD patterns of fresh Gd-B after MeOH release.

Supplementary Table 1 | Synthetic conditions for Gd-B single-crystal.

<table>
<tr><td>Entry</td><td colspan="3">Solution 1</td><td colspan="4">Solution 2</td></tr>
<tr><td></td><td>$C(NH_2)_3Cl$</td><td>MeOH</td><td>H_2O</td><td>H_3BO_3</td><td>MeOH</td><td>H_2O</td><td>TEA (mL)</td></tr>
<tr><td>A1</td><td rowspan="10">1.86 g</td><td rowspan="5">21 mL</td><td rowspan="5">/</td><td rowspan="10">600 mg</td><td rowspan="5">24 mL</td><td rowspan="5">/</td><td>0</td></tr>
<tr><td>A2</td><td>0.169</td></tr>
<tr><td>A3</td><td>0.338</td></tr>
<tr><td>A4</td><td>0.675</td></tr>
<tr><td>A5</td><td>1.350</td></tr>
<tr><td>B1</td><td rowspan="5">/</td><td rowspan="5">21 mL</td><td rowspan="5">/</td><td rowspan="5">24 mL</td><td>0</td></tr>
<tr><td>B2</td><td>0.169</td></tr>
<tr><td>B3</td><td>0.338</td></tr>
<tr><td>B4</td><td>0.675</td></tr>
<tr><td>B5</td><td>1.350</td></tr>
</table>

The tetrahedral size and amount of Gd-B single crystal is largely dependent on TEA volume, as entries A1-A3 only give trace amount of floc or crystal product and A5 shows higher amount and larger crystal size than that of A4.

Supplementary Table 2 | Elemental analysis of Gd-B.

Sample	C (wt%)	N (wt%)	H (wt%)
Gd-B	28.61	19.94	9.32
Theoretical	29.72	20.79	9.40

Supplementary Table 3 | Comparison of MeOH adsorption capability among various adsorbents.

Material	Adsorption amount (g/g)	Ref
Gd-B*	0.60 (417 cm^3/g)	this work
UiO-67	0.34	2
ZIF-8(Zn)	0.37	3
MIL-53(Cr)	0.53	4
MIL-100(Cr)	0.67	5
HKUST-1	0.50	6
Al(OH)-(1,4-NDC)	0.16	7
Zn_2(bptc)	0.10	8

*The adsorption amount of air-dried sample includes the physical adsorption of MeOH and MeOH adsorption for the formation of borate to re-construct Gd-B framework.

Supplementary References

1. Steinberg, H. Boron-oxygen and boron-sulfur compounds. *Organoboron Chemistry*, Volume 1. Interscience Publishers, John Wiley and Sons, Inc., New York, 1964.

2. Katz, M. J., et al. A facile synthesis of UiO-66, UiO-67 and their derivatives. Chem. Commun. **49**, 9449-9451 (2013).

3. Park, K. S., et al. Exceptional chemical and thermal stability of zeolitic imidazolate frameworks. Proc. Natl. Acad. Sci. U. S. A. **103**, 10186-10191 (2006).

4. Bourrelly, S, et al. Exploration of the adsorption of polar vapors in the highly flexible metal organic framework MIL-53(Cr). J. Am. Chem. Soc. **132**, 9488-9498 (2010).

5. Fef́ey, G., et al. A hybrid solid with giant pores prepared by a combination of targeted chemistry, simulation, and powder diffraction. Angew. Chem., Int. Ed. **43**, 6296-6301 (2004).

6. Jeremias, F., et al. Water and methanol adsorption on MOFs for cycling heat transformation processes. New J. Chem. **38**, 1846-1852 (2014).

7. Comotti, A., et al. Nanochannels of two distinct cross-sections in a porous Al-based coordination polymer. J. Am. Chem. Soc. **130**, 13664-13672 (2008).

8. Lin, X., et al. A porous framework polymer based on a zinc(II) 4,4'-bipyridine-2,6,2',6'-tetracarboxylate: synthesis, structure, and "zeolite-like" behaviors. J. Am. Chem. Soc. **128**, 10745-10753 (2006).
