# Supplementary material for: Combustible ice mimicking behavior of hydrogen-bonded organic framework at ambient condition
Source: Nat Commun. 2020 Jun 19;11:3124. doi: 10.1038/s41467-020-16976-1 (PMC7305155; doi:10.1038/s41467-020-16976-1)
Supplement: Supplementary file 3 — Description of Additional Supplementary Files [file 41467_2020_16976_MOESM3_ESM.pdf]

**Description of Additional Supplementary Files**

File Name: Supplementary Movie 1
Description: Accommodated MeOH molecules in Gd-B released in air for lighting.
